# Assessment of the Impact of Treadmill Training with Digital Biofeedback on Functional Performance and Gait Parameters of Patients After Total Hip Replacement—A Randomized Study

**DOI:** 10.3390/jcm15062314

**Published:** 2026-03-18

**Authors:** Aleksandra Milewska, Agnieszka Przedborska, Robert Irzmański

**Affiliations:** Department of Internal Diseases, Rehabilitation and Physical Medicine, Medical University of Lodz, 90-647 Lodz, Poland; agnieszka.przedborska@umed.lodz.pl (A.P.); robert.irzmanski@umed.lodz.pl (R.I.)

**Keywords:** hip replacement, total hip arthroplasty, rehabilitation, gait analysis, gait speed, biofeedback

## Abstract

**Background**: Gait re-education is one of the key elements of comprehensive rehabilitation after total hip replacement. Recent technological advancements allow patients to benefit from increasingly sophisticated training solutions based on biofeedback. The aim of this study was to assess the impact of a treadmill training protocol with a digital biofeedback component on the gait parameters of patients after an uncomplicated total hip replacement and on their functional status. **Methods**: The study included 137 patients after total hip replacement. In the control group, traditional walking training with biofeedback in the form of a mirror was used. In the study group, the Biodex Gait Trainer 3 treadmill was used for this purpose, which also served as a diagnostic tool for both groups. The following parameters were assessed: distance, average walking speed, average step cycle, step length, coefficient of variability and time on each foot. Additionally, the study included the Timed Up & Go (TUG) test and the use of orthopedic supplies. Results were considered statistically significant at *p* < 0.05. **Results**: Significant statistical differences were found between the groups in terms of distance, average walking speed, and step length. Additionally, significantly shorter TUG times were observed and a higher rate of discontinuation of orthopedic supplies in the study group. However, the therapy method did not have a significant effect on the average step cycle, coefficient of variability or time on each foot. These parameters showed comparable improvement in both groups. **Conclusions**: Treadmill training with visual biofeedback has a positive effect on certain gait parameters. The greatest benefits from this type of training can be gained by patients with deficits in stability and mobility in space.

## 1. Introduction

Hip osteoarthritis is one of the most common causes of physical disability in the adult population [1]. The progressive degenerative changes in the intra-articular structures and the surrounding soft tissues lead to significant pain, stiffness, and limitation of range of motion in the affected joint [2,3]. This leads to reduced independence in daily life and a more frequent need for healthcare services [4].

Therapeutic management of hip osteoarthritis varies depending on the severity of degenerative changes; in the advanced stages of the disease, total hip arthroplasty is indicated [1]. However, a patient’s return to full functional capacity depends on comprehensive and individualized physiotherapeutic management implemented both before and after surgery [4]. One of its key components is gait re-education and the restoration of appropriate limb loading conditions during ambulation in order to prevent damage resulting from systematic overloading of the unaffected joint [3]. The safe ambulation in a complex and changing environment leads to greater independence and reduces the risk of falls [5,6]. It involves varying walking speed and distance, as well as the ability to combine walking with other tasks requiring cognitive engagement [6].

In this latter aspect, various modern technological solutions based on the biofeedback mechanism may be applied. Biofeedback relies on a simple and effective feedback loop through which the patient discerns differences between their intentions and the actual outcomes of their training efforts [7,8].

An example of such a diagnostic and training device is the Biodex Gait Trainer™ treadmill, designed for neurological, orthopedic, and geriatric patients. Equipped with load cells at each corner, it allows precise step detection. During training, the patient receives real-time feedback on the actual step length, while a metronome provides the desired step rhythm. At the same time, numerous additional parameters describing locomotion are assessed, not just speed and transit time. The absence of such detailed analysis is often considered as a significant limitation of many studies [9].

The present study focuses on assessing the impact of treadmill training, with an added element of primarily digital biofeedback, on gait kinematic parameters and the functional status of patients following total hip replacement.

## 2. Materials and Methods

### 2.1. Ethics Statement

The study received a positive opinion from the Bioethics Committee at the Medical University of Lodz, approval no. RNN/118/24/KE, dated 14 May 2024. The research was conducted as part of the institution’s statutory activities, and each participant provided informed consent. At every stage, patients had the option to withdraw from the study without any consequences, while retaining the right to continue receiving medical care.

### 2.2. Characteristics of Study Participants

Between May and October 2025, 137 patients (68 women and 69 men) participated in the study at the Clinic of Orthopedic and Post-Traumatic Rehabilitation, University Clinical Hospital No. 2 in Lodz. The mean age of the participants was 67.42 ± 11.07 years, with half of the patients being 69 years or younger (Q25–Q75: 59–76 years). The average time since surgery was 2.56 ± 0.47 months, and for half of the participants, it did not exceed 3 months (Q25–Q75: 2–3 months).

### 2.3. Inclusion and Exclusion Criteria

The inclusion criteria consisted of an uncomplicated total hip arthroplasty due to advanced osteoarthritis and the patient’s consent to participate in the training procedure. The exclusion criteria included a complicated postoperative course (thromboembolic events, perioperative infections, or a history of endoprosthesis dislocation), participation in rehabilitation using other modern biofeedback-based therapeutic methods within the six months prior to admission to the clinic, and refusal to participate in the study.

### 2.4. Study Design

For the purpose of verification of potential candidates for the study, a protocol was developed which, in addition to basic demographic data, included the Timed Up & Go (TUG) test, utilized to assess the participants’ functional status, specifically mobility, balance in space, and potential fall risk. The test procedure consisted of standing up from a chair, walking a distance of 3 m, turning around, returning to a seated position on the chair [10]. Before the test, the patient was provided with all necessary instructions. The test was performed three times, and the best result was included in the final analysis.

The Biodex Gait Trainer 3 (Biodex Medical Systems, Shirley, NY, USA) was used in the study as both a therapeutic and diagnostic device. During the 10 min treadmill walking test, using the integrated strain gauges embedded in it, the following parameters were recorded: distance (m), average walking speed (m/s), average step cycle (cycles/s), and—separately for both lower extremities—step length (cm), time on each foot (%), and the coefficient of variation (%) [11]. It represents the percentage value reflecting the degree of irregularities occurring between consecutive steps [12]. The main result of this study was a change in the average walking speed and step length, which are among the key values affecting locomotion efficiency. The other walking parameters were treated as secondary outcomes.

The design of this two-arm study involved the random and blinded allocation of participants into two groups following the baseline assessment conducted in accordance with the described protocol. The investigators had no influence on the assignment of individual patients to the study groups. A random number generator was used in the group assignment procedure with a biased coin design to eliminate inequalities between the groups, particularly their sizes. The nature of the study made it impossible to implement double-blinding, which may constitute a significant limitation.

The study group represented 70 participants (51.1%), while the control group included 67 participants (48.9%). A comparative baseline characteristic of both groups is presented in Table 1.

There were no significant differences between the groups with respect to age, sex, body mass index (BMI), time elapsed since surgery, operated limb, or type of orthopedic management.

The mean age in the study group was 67.17 ± 10.41 years old, and in the control group 67.67 ± 11.79 years old. Both groups had a balanced sex structure. The mean body mass index (BMI) was 28.41 ± 4.17 kg/m^2^ in the study group and 28.54 ± 4.45 kg/m^2^ in the control group. In both groups, the majority of participants were overweight (48.6% in the study group and 50.8% in the control group, respectively).

The time since surgery did not exceed 2.75 months for half of the patients in the study group (Q25–Q75: 2–3 months) and 3 months in the control group (Q25–Q75: 2–3 months). In the study group, more patients had surgery on the right limb (57.1%), whereas in the control group, the majority had surgery on the left limb (55.2%).

In both the study and control groups, the majority of participants did not require orthopedic support, accounting for 51.4% and 55.2% of patients, respectively.

In the control group, gait retraining was conducted on a traditional course with additional handrails and obstacles, and with biofeedback provided in the form of a mirror [13]. Gait training was performed on parallel bars, using a standardized obstacle course that consisted of three obstacles placed along a 3 m walking path. The obstacles included low wooden blocks with a height of 10–15 cm, and the distance between them was approximately 60–80 cm. Participants were instructed to walk at a comfortable pace, overcoming the obstacles. The physiotherapist supervised the task, ensured safety, and provided professional support. A full-length mirror (approximately 150 × 50 cm) was placed about 1.5 m from the parallel bars in front of the participant. Participants were instructed to observe their body posture and lower limb movements while walking. Each patient was tasked with independently controlling their body posture and the correctness of their steps using their reflection in a mirror, whereas the study group underwent gait retraining using the Biodex Gait Trainer 3 (Biodex Medical Systems, Inc., Shirley, NY, USA) with biofeedback provided via an application that allowed real-time monitoring of gait parameters. The other components of the training (isometric exercises of the quadriceps, gluteal muscles, and lower limb rotators) were identical in both groups. The training was conducted for a three-week period during the patient’s hospital stay, with a weekend break.

In both groups, the initial duration of gait training was 10 min; after a five-day individual adaptation based on blood pressure and heart rate values, it increased to 30 min. In this way, patients in both groups completed 5 adaptation training sessions and 10 actual sessions.

On the final day of the hospital stay, an end-of-study assessment was conducted to evaluate the outcomes using the established protocol. Each assessment was performed by the same therapist to eliminate potential measurement errors.

### 2.5. Statistical Methods

Quantitative variables were described using the mean and standard deviation (SD), ordinal measures including the median (Me) and quartiles (Q25 and Q75), as well as the minimum and maximum values (Min–Max). The normality of the variables was assessed using the Shapiro–Wilk test. For categorical variables, the number of observations for a given category and the corresponding percentage were reported.

To compare two independent groups, student’s *t*-test was used when the variables were normally distributed in both groups, and the nonparametric Mann–Whitney U test was applied in cases of non-normal distribution.

For qualitative variables, group comparisons were performed using the chi-square test of independence and the McNemar–Bowker’s test.

For comparisons between groups with repeated measurements, due to unmet assumptions (the normality of the distribution of the variables, equality of variances, sphericity), a two-way nonparametric analysis of variance was used—specifically, the ART ANOVA (Aligned Rank Transformation ANOVA). This method aligns and ranks data before applying ANOVA procedures, allowing nonparametric analysis of factorial designs while preserving interaction effects. Compared with traditional nonparametric tests, ART ANOVA allows the analysis of interaction effects in multifactorial designs. Post hoc comparisons were performed using Tukey’s test with Bonferroni correction. Additionally, the partial η^2^ was reported as a measure of effect size (η^2^ ≈ 0.01 indicates a small effect, η^2^ ≈ 0.06 a medium effect, and η^2^ ≈ 0.14 or higher a large effect). Results were considered statistically significant at *p* < 0.05. All calculations were performed using the PQStat software package v. 1.8.6 and the R environment version 4.3.2 (ARTool package v0.11.1).

## 3. Results

In the research, the time achieved in the TUG test before and after therapy was compared between the groups (Table 2). A statistically significant interaction effect was observed (*p* < 0.0001), indicating that TUG results differed depending on the therapy method. In the study group, TUG time decreased significantly after therapy (*p* < 0.0001), with a mean value of 12.46 ± 3.78 s before therapy and 9 ± 2.89 s after therapy. Also, the control group showed a significant reduction in TUG time after therapy (*p* < 0.0001). Before therapy, the mean value was 13.34 ± 4.3 s, and after therapy it decreased to 11.67 ± 3.78 s. Notably, post-therapy TUG times were significantly lower in the study group compared to the control group (*p* < 0.0001), with mean values of 9 ± 2.89 s in the study group and 11.67 ± 3.78 s in the control group.

Table 3 presents the results of the comparison between patients from both groups with respect to distance, average walking speed, and average step cycle before and after therapy.

With regard to the distance covered, a statistically significant interaction effect was observed (*p* < 0.0001), indicating that the distance walked during the walking test differed depending on the training method applied. In the study group, the mean distance before therapy was 255.8 ± 92.87 m, and after therapy the parameter increased to 536.51 ± 182.81 m; this effect should be considered large (*p* < 0.0001). In the control group, an increase in the mean distance was also observed, from 271.69 ± 129.12 m before therapy to 388.75 ± 163.68 m after the therapy. However, the distance covered after therapy was significantly longer in the study group (*p* < 0.0001). No statistically significant differences between the groups were observed before therapy (*p* = 1.0000).

Average walking speed was also analyzed. In the study group, the average walking speed was 0.45 ± 0.17 m/s before therapy and 0.88 ± 0.37 m/s after therapy. In the control group, it was 0.45 ± 0.20 m/s before therapy, increasing to 0.66 ± 0.24 m/s after therapy.

In contrast, no statistically significant differences between the groups were found for the average step cycle, either before or after therapy. In the study group, the average step cycle was 0.59 ± 0.11 before therapy and increased to 0.71 ± 0.14 after therapy. In the control group, this parameter was 0.59 ± 0.14 before therapy and 0.75 ± 0.64 after 3 weeks of rehabilitation. An increase in this parameter was observed in both groups after the therapy; however, the change was not statistically significant (*p* = 0.0751).

Additionally, the study compared specific gait parameters such as step length, coefficient of variability and time on each foot for operated and unaffected limb.

For the step length, a statistically significant interaction effect was observed (*p* < 0.0001) for both the operated and non-operated limbs (Table 4). In the study group, before the therapy, the step length was 33.41 ± 10.65 cm for the operated limb and 33.69 ± 10.95 cm for the non-operated limb; after the therapy, the values increased to 56.7 ± 10.84 cm for the operated limb and 56.81 ± 10.8 cm for the non-operated limb. In both cases, the effect size was considered large (*p* < 0.0001). In the control group, after the therapy, the step length of the operated limb increased significantly from 35.22 ± 11.96 cm to 45.91 ± 12.85 cm (*p* < 0.0001). A similar result was observed for the non-operated limb, with the parameter increasing from 35.55 ± 11.96 cm to 46.49 ± 12.88 cm after the therapy. The step length for both the operated and non-operated limbs was significantly greater in the study group after the therapy (*p* < 0.0001). No statistically significant differences were observed between the groups prior to therapy regarding the step length of either limb (*p* = 1.0000).

In contrast, the interaction effect for the coefficient of variation was not statistically significant for either the operated limb (*p* = 0.2677) or the unaffected one (*p* = 0.6452) (Table 5). However, it can be noted that in both groups, post-therapy values were lower than those recorded before the therapy. The main effect of time in the analysis of variance was statistically significant for the operated limb (*p* < 0.0001; partial η^2^ = 0.56) as well as for the unaffected limb (*p* < 0.0001; partial η^2^ = 0.58). Thus, the coefficient of variation decreased after the therapy, regardless of the treatment method.

Similarly, the interaction effect for time on each foot was not statistically significant for either the operated limb (*p* = 0.2655) or the unaffected one (*p* = 0.2282) (Table 6). The therapy method did not influence changes in this parameter.

To provide a more comprehensive assessment of the functional status of the patients, the need for and extent of additional functional aids in the form of orthopedic devices were analyzed (Table 7).

No statistically significant differences were observed between the groups in terms of the type of orthopedic device prior to the therapy. However, a significant difference was noted after the therapy (*p* = 0.0046), with a markedly higher proportion of patients in the study group not requiring any orthopedic supplies (87.1% vs. 68.7%). In both groups, a statistically significant increase was observed post-therapy in the proportion of patients not using additional functional aids.

## 4. Discussion

Gait is a key functional ability essential for optimal daily functioning, and its rehabilitation following surgical procedures is a vital component of the patient’s return to full functional capacity.

The present study is an extension of the pilot experiment [14] and was conducted on a larger group of volunteers. Furthermore, it incorporates a greater number of parameters for analysis and compares treadmill-based training with traditional gait rehabilitation methods. It should be noted that the use of a treadmill as part of post-operative rehabilitation following total hip arthroplasty is not innovative and has been applied for many years [15,16]. Apart from the aforementioned pilot study, no reports were found on the use of the Gait Trainer 3 treadmill to recreate normal gait conditions in the group of patients under analysis. However, the equipment used in this study is widely applied in the diagnosis and rehabilitation of patients with other conditions, particularly neurological and cardiological disorders [11,12,17,18]. Therefore, it appears to be a reliable measurement tool.

The aim of this study was to test the hypothesis that treadmill training with digital biofeedback provides greater benefits compared to traditional gait rehabilitation using an obstacle course with mirror biofeedback.

The analysis primarily focused on basic gait parameters, namely the distance covered during a 10 min walk and the average walking speed. Both measures showed significant changes and differed depending on the rehabilitation method. After a series of treadmill training sessions, they improved to a much greater extent, contrary results were obtained by Hesse et al. [15]. This may be due to the older publication date of the cited study and the technological advancements that have occurred in recent years. However, the fact of constant and precise speed control while walking on a treadmill, compared to self-monitoring during traditional training, could have influenced the achievement of such results.

Walking speed is an important aspect of locomotion in relation to more precise gait parameters, as it has been shown to have a direct influence on them [19]. The systematic review by Fukuchi et al. [19] highlights the relationship between the walking speed and the step length, a finding that was also confirmed in the present study. In the group undergoing treadmill training, a statistically significant increase in step length was observed for both the operated and non-operated limbs. This effect may be due to the type of feedback used in both trainings. Digital biofeedback provided the possibility of continuous and precise monitoring of the length of each step taken, compared to mirror biofeedback where the responsibility for control and correction rested solely on the patient and therapist. The cited study also indicates favorable changes in hip and knee joint kinematics at higher walking speeds, particularly regarding hip flexion and knee extension movements. Other studies have also reported similar trends regarding angular velocities of the lower limb joints [20,21,22]. Therefore, walking speed and its precise modulation during training may positively influence the rehabilitation of joint range of motion in the lower limbs, for example following extensive surgical procedures.

A parameter directly related to the angular movement of the lower limb joints during gait is the step cycle, defined as the time between two consecutive heel strikes of the same foot [23]. It would be expected that its values would change with increasing walking speed. However, the present study yielded opposite results, with no statistically significant changes observed in this parameter for either of the analyzed groups. This finding is consistent with available literature, which were conducted on different patient populations [24].

To further investigate locomotor function following extensive total hip arthroplasty, additional parameters were included, namely the coefficient of variation and the time on each foot. Both measures improved regardless of the rehabilitation method, indicating a reduction in the coefficient of variation and a more balanced distribution of weight-bearing time between the feet. There are no studies that have specifically analyzed these parameters in orthopedic patients. However, a study reporting opposite findings was conducted in a population with neurological disorders, making direct comparison with the present results inappropriate [25]. The large number of results from the initial study in both groups, which were close to the norm, could have influenced the obtained results.

Limitations in mobility resulting from an inefficient gait pattern significantly reduce daily physical and social activity, directly contributing to loss of independence and autonomy [12,26,27]. Patients particularly at risk of this phenomenon are those who have undergone extensive surgical procedures on the lower limbs and spine, as well as elderly people with multiple comorbidities. For this reason, it was decided to analyze two additional aspects in the study describing patients’ self-sufficiency and spatial mobility: the Timed Up and Go (TUG) test and the need for orthopedic aids in the form of one or two elbow crutches. After three weeks of training, both measures showed favorable changes in both observed groups. However, the experimental group demonstrated significantly lower TUG test times and more frequent discontinuation or reduction in the use of orthopedic aids compared to the control group, a pattern also reported by other authors studying various conditions in patient populations [17,28,29]. Translating these findings into clinical practice, treadmill training with biofeedback can be safely implemented as a key component of functional rehabilitation and an effective fall-prevention strategy in patients after the orthopedic surgery and in older adults, as high values achieved in the mentioned test are directly related to fall history [30].

The effectiveness of the gait rehabilitation protocol examined in this study may be attributed to the difference in training conditions, namely a moving treadmill versus a stationary surface. Numerous studies indicate surface instability as a key distinction between the compared conditions [31]. Such sensory feedback may contribute to emphasizing postural stability during walking [32,33,34].

Treadmill training with digital biofeedback is fully aligned with the concept of modern postoperative rehabilitation due to its capacity for flexible, individualized load adjustment and continuous control of training conditions. Based on the obtained results, it appears to be an effective alternative to traditional gait rehabilitation in certain aspects, particularly in terms of spatial mobility. However, considerably more research is needed in this area to compare outcomes in larger groups of patients, including those with other orthopedic conditions and over longer periods of time to verify long-term therapeutic effects. The present study represents one of the first reports of this kind and does not provide sufficient evidence to unequivocally establish the superiority of treadmill gait training over traditional rehabilitation or of one form of biofeedback over another. An important issue that remained unresolved in the study is the lack of an objective and precise measurement method for the training intensity of individual walking sessions on the obstacle course. This should be regarded as its significant limitation. Other shortcomings are single-center nature of the study and the inability to blind the physiotherapist conducting the training sessions and the patients in both groups, which could have affected the results obtained. Future studies should also incorporate assessments of subjective quality of life and scales focusing on the patient perspective, which can provide a broader picture of the topic being discussed.

## 5. Conclusions

The treadmill training with visual biofeedback positively affects certain gait parameters, including distance, average walking speed, and step length. However, no significant improvements were observed in average step cycle, coefficient of variation, or the time on each foot. In terms of functional outcomes, assessed by the TUG test and the need for using elbow crutches, a more favorable effect of treadmill exercises compared to standard intervention was observed. This may directly translate into a reduction in the risk of falls.

The greatest benefits from this type of training may be observed in patients with deficits in stability and spatial mobility. In patients following orthopedic surgery, it can be incorporated as a complementary component of a standard therapy to further enhance rehabilitation outcomes.

## Figures and Tables

**Table 1:** Characteristics of the compared groups.

Variable		Measures	Group		*p*-Value
			Study Group (n = 70)	Control Group (n = 67)	
Age		Mean ± SD	67.17 ± 10.41	67.67 ± 11.79	0.6018
		Me [Q25; Q75]	68.5 [59.75; 75]	69 [59.5; 77]	
		Min; Max	38; 87	32; 89	
Gender	F	n (%)	35 (50%)	33 (49.25%)	0.9304
	M		35 (50%)	34 (50.75%)	
BMI		Mean ± SD	28.41 ± 4.17	28.54 ± 4.45	0.8554
		Me [Q25; Q75]	27.63 [25.53; 31.78]	28.44 [26;31.23]	
		Min; Max	20.42; 37.33	16.9; 43.9	
BMI	norm	n (%)	13 (18.57%)	12 (17.91%)	0.9679
	overweight		34 (48.57%)	34 (50.75%)	
	obesity		23 (32.86%)	21 (31.34%)	
Time (months)		Mean ± SD	2.56 ± 0.47	2.56 ± 0.47	0.9714
		Me [Q25; Q75]	2.75 [2; 3]	3 [2; 3]	
		Min; Max	2; 3	2; 3	
Operated limb	R	n (%)	40 (57.14%)	30 (44.78%)	0.1478
	L		30 (42.86%)	37 (55.22%)	
Orthopedic supplies	1 crutch	n (%)	16 (22.86%)	13 (19.4%)	0.8663
	2 crutches		18 (25.71%)	17 (25.37%)	
	none		36 (51.43%)	37 (55.22%)	

**Table 2:** The Timed Up and Go (TUG) test results before and after therapy in the compared groups.

Group	Measures	Before Therapy	After Therapy	*p*-Value for ANOVA (Group × Time Interaction Effect)	Partial η^2^
Study group	Mean ± SD	12.46 ± 3.78	9 ± 2.89	<0.0001	0.21
	Me [Q25; Q75]	12.08 [10; 14.25]	8.43 [6.86; 10.45]		
	Min; Max	5.46; 28.42	4.71; 19.39		
Control group	Mean ± SD	13.34 ± 4.3	11.67 ± 3.78		
	Me [Q25; Q75]	12.55 [10.12; 15.27]	10.95 [8.78; 14.07]		
	Min; Max	7.16; 27.79	5.07; 22.98		

**Table 3:** Distance, average walking speed and average step cycle before and after therapy in the compared groups.

Variable	Group	Measures	Before Therapy	After Therapy	*p*-Value for ANOVA (Group × Time Interaction Effect)	Partial η^2^
Distance (m)	Study group	Mean ± SD	255.8 ± 92.87	536.51 ± 182.81	<0.0001	0.24
		Me [Q25; Q75]	246.5 [200.25; 312.75]	536 [416; 655.75]		
		Min; Max	60; 467	141; 1081		
	Control group	Mean ± SD	271.69 ± 129.12	388.75 ± 163.68		
		Me [Q25; Q75]	242 [176; 355]	362 [267; 492.5]		
		Min; Max	39; 581	43; 805		
Average walking speed (m/s)	Study group	Mean ± SD	0.45 ± 0.17	0.88 ± 0.37	<0.0001	0.17
		Me [Q25; Q75]	0.43 [0.34; 0.53]	0.84 [0.68; 1.02]		
		Min; Max	0.19; 1.2	0.24; 2.9		
	Control group	Mean ± SD	0.45 ± 0.2	0.66 ± 0.24		
		Me [Q25; Q75]	0.4 [0.31; 0.54]	0.63 [0.48; 0.82]		
		Min; Max	0.16; 1.01	0.27; 1.34		
Average step cycle (cycle/s)	Study group	Mean ± SD	0.59 ± 0.11	0.71 ± 0.14	0.0751	0.02
		Me [Q25; Q75]	0.59 [0.53; 0.65]	0.73 [0.62; 0.82]		
		Min; Max	0.34; 0.89	0.4; 1.11		
	Control group	Mean ± SD	0.59 ± 0.14	0.75 ± 0.64		
		Me [Q25; Q75]	0.58 [0.52; 0.69]	0.65 [0.59; 0.75]		
		Min; Max	0.28; 1	0.47; 5.82		

**Table 4:** Step length (cm) before and after therapy in the compared groups.

Limb	Group	Measures	Before Therapy	After Therapy	*p*-Value for ANOVA (Group × Time Interaction Effect)	Partial η^2^
Operated Limb	Study group	Mean ± SD	33.41 ± 10.65	56.7 ± 10.84	<0.0001	0.24
		Me [Q25; Q75]	32 [25.25; 41.75]	56 [52; 64.5]		
		Min; Max	12; 61	24; 77		
	Control group	Mean ± SD	35.22 ± 11.96	45.91 ± 12.85		
		Me [Q25; Q75]	34 [27; 43]	45 [38; 56.5]		
		Min; Max	13; 62	19; 68		
Unaffected Limb	Study group	Mean ± SD	33.69 ± 10.95	56.81 ± 10.8	<0.0001	0.21
		Me [Q25; Q75]	33 [24; 41]	56 [52; 64.75]		
		Min; Max	12; 60	23; 77		
	Control group	Mean ± SD	35.55 ± 11.96	46.49 ± 12.88		
		Me [Q25; Q75]	35 [28; 43.5]	46 [37.5; 57]		
		Min; Max	13; 64	17; 68		

**Table 5:** Coefficient of variation (%) before and after therapy in the compared groups.

Limb	Group	Measure	Before Therapy	After Therapy	*p*-Value for ANOVA (Group × Time Interaction Effect)	Partial η^2^
Operated Limb	Study group	Mean ± SD	16.03 ± 15.68	4.64 ± 2.27	0.2677	0.10
		Me [Q25; Q75]	13 [6; 19.75]	4 [3; 4.75]		
		Min; Max	4; 107	3; 13		
	Control group	Mean ± SD	17.06 ± 17.6	8.24 ± 6.21		
		Me [Q25; Q75]	12 [8; 19.5]	6 [4; 10]		
		Min; Max	3; 108	3; 33		
Unaffected Limb	Study group	Mean ± SD	15.94 ± 14.99	4.81 ± 2.77	0.6452	0.001
		Me [Q25; Q75]	12 [7; 17]	4 [3; 5]		
		Min; Max	4; 99	3; 15		
	Control group	Mean ± SD	16.72 ± 14.57	8.27 ± 6.91		
		Me [Q25; Q75]	11 [7.5; 21.5]	6 [4; 9]		
		Min; Max	4; 85	3; 43		

**Table 6:** Time on each foot (%) before and after therapy in the compared groups.

Limb	Group	Measure	Before Therapy	After Therapy	*p*-Value for ANOVA (Group × Time Interaction Effect)	Partial η^2^
Operated Limb	Study group	Mean ± SD	49.9 ± 2.96	50.16 ± 1	0.2655	0.01
		Me [Q25; Q75]	50 [49; 51]	50 [50; 50]		
		Min; Max	43; 59	48; 53		
	Control group	Mean ± SD	49.9 ± 2.32	49.63 ± 2.17		
		Me [Q25; Q75]	50 [48; 51]	50 [49; 51]		
		Min; Max	43; 57	43; 55		
Unaffected Limb	Study group	Mean ± SD	50.1 ± 2.96	49.83 ± 1.02	0.2282	0.01
		Me [Q25; Q75]	50 [49; 51]	50 [50; 50]		
		Min; Max	41; 57	47; 52		
	Control group	Mean ± SD	50.09 ± 2.31	50.37 ± 2.17		
		Me [Q25; Q75]	50 [49; 51.5]	50 [49; 51]		
		Min; Max	43; 57	45; 57		

**Table 7:** Orthopedic supplies before and after therapy in the compared groups.

Time	Orthopedic Supplies	Group		*p*-value (Before vs. After Therapy)
		Study Group (n = 70)	Control Group (n = 67)	
Before Therapy	1 crutch	16 (22.86%)	13 (19.4%)	0.8663
	2 crutches	18 (25.71%)	17 (25.37%)	
	none	36 (51.43%)	37 (55.22%)	
After Therapy	1 crutch	9 (12.86%)	13 (19.4%)	0.0046
	2 crutches	0 (0%)	8 (11.94%)	
	none	61 (87.14%)	46 (68.66%)	
*p*-value (comparison of groups)		<0.0001	0.0129	x

## Data Availability

The original contributions presented in this study are included in the article. Further inquiries can be directed to the corresponding author.
